# A Unique Case Report on Methadone Used for Treatment of Opioid-induced Hyperalgesia in a Cancer Patient at the End of Life

**DOI:** 10.7759/cureus.5394

**Published:** 2019-08-15

**Authors:** Heather N Bitar, Andre M Cipta, Kuo-Wei Lee, Wesley S Woo

**Affiliations:** 1 Supportive Medicine, City of Hope, Duarte, USA; 2 Geriatrics, Palliative Medicine, and Continuing Care, Kaiser Permanente, Los Angeles, USA

**Keywords:** opioid induced hyperalgesia, methadone, opioid tolerance, opioid dependence, dilaudid, cancer pain, end of life care, mu opioid receptor, nmda receptor

## Abstract

Opioids are the cornerstone of palliative pain management. Opioids work on the mu-opioid receptor as an agonist for the treatment of pain. Repeated exposure to opioids over time can lead to undesired desensitization of the antinociceptive receptor while sensitizing the N-methyl-D-aspartate (NMDA) pathway, causing a paradoxical effect where the treatment of pain creates more sensitivity to certain stimuli. This phenomenon is known as opioid-induced hyperalgesia (OIH). Methadone, a synthetic opioid, may be more effective for pain and offers advantages over other opioids in specific clinical situations due to its partial antagonistic effect on the NMDA pathway. We describe a unique case where as needed (prn) and continuous intravenous (IV) methadone was effective in relieving OIH caused by high doses of IV Dilaudid for intractable cancer pain at the end of life. Given its unique pharmacokinetics, effective pain control, and the prevention of suffering from OIH, methadone should be considered earlier on in palliative pain management, especially in those patients predicted to require high levels of opioid dosing.

## Introduction

Opioids remain the cornerstone of palliative pain management due to their rapid onset and flexible dosing. Opioids mainly work on the mu-opioid receptor as an agonist for the treatment of pain. Repeated exposure to opioids over time can lead to undesired desensitization of the antinociceptive receptor requiring escalation in opioid doses leading to the development of tolerance. At times, for exact mechanisms unknown and multiple theories being hypothesized, this leads to sensitization of the pronociceptive N-methyl-D-aspartate (NMDA) receptor pathway as a compensatory response to opioid receptor stimulation. Repeated exposure to opioids over time in some patients can cause a paradoxical effect where the treatment of pain creates more sensitivity to certain stimuli [1]. This phenomenon is known as opioid-induced hyperalgesia (OIH).

Methadone, a synthetic opioid, is a mu-opioid receptor agonist and NMDA receptor antagonist. The use of methadone may be more effective and can offer advantages for palliative pain management near the end of life where high dose opioids may be necessary. We describe a unique case where intravenous (IV) methadone used as a continuous infusion and as needed (prn) with specific dosing was effective in relieving OIH in a patient experiencing inadequate pain control and dose limiting side effects from high dose IV Dilaudid for cancer-associated pain at the end of life.

## Case presentation

A 56-year-old man was diagnosed with metastatic pancreatic cancer to the liver, lung, and duodenum in April of 2017. He had a gastrostomy tube placed for venting due to malignant bowel obstruction and a biliary drain due to invasion into the biliary tree. He also had a peripherally inserted central catheter (PICC) placed to provide continuous total parental nutrition (TPN), as he was malnourished from intractable abdominal pain, nausea, and vomiting every time he ate. He had attempted chemotherapy but stopped due to intolerance of its side effects. His pain regimen when on home palliative care was fentanyl 250 mcg/hr patch every 72 hrs and one to two tablets of Dilaudid 4 mg by mouth every four hours as needed, which he eventually required around the clock.

Pain management

His pain continued to worsen and after he started hospice care in September of 2017. He was placed on an IV continuous Dilaudid infusion by continuous ambulatory delivery device (CADD) pump with prn IV Dilaudid administered by patient controlled administration (PCA) because he became too cachectic to have reliable delivery of fentanyl with multiple patches. He was started on Dilaudid 1 mg/hr with 2 mg q15min prn breakthrough pain, which was increased weekly by 50%-100% due to inadequate pain control. He was also on adjunctive medications at various points throughout his care including a serotonin-norepinephrine reuptake inhibitor (SNRI), gabapentin, steroids, lorazepam, and octreotide. He was offered but declined a celiac plexus block. Concurrently, he was receiving non-pharmacological management of counseling and psychological support for anticipatory grief through our clinical licensed social worker and nurse case manager.

In the last two weeks of his life, he required exponential amounts of Dilaudid up to 30 mg/hr with 15 mg prn q15min for breakthrough, which seemed to be making his pain worse. At one point he or his family clicked the PCA button 107 times in a 24-hour period with worsening symptoms such as progression to back pain, agitation, delirium, and myoclonus.

Differential diagnosis

In a patient with metastatic cancer with chronic pain and elevated levels of distress, there is a wide range of diagnoses to consider. This includes but is not limited to opioid tolerance, opioid dependence, worsening of disease such as metastasis to the brain, malfunction or deep venous thrombosis (DVT) occluding the PICC line affecting delivery of Dilaudid, metabolic derangements, hypoxia, and infection. Often, pain tolerance will present as an increase in pain in the previously localized area and gradually responds appropriately to an increase in dose of pain medication [2]. This is a key differential marker from OIH where pain distribution spreads, or worsens with increase in pain medication. Opioid tolerance is defined as the need for an increased dose of pain medications to have the same effect on pain control. This is a physiologic phenomenon occurring in all patients with chronic pain treated with opioid pain medications. Tolerance leads to higher opioid dosing to achieve adequate analgesia, putting patients at risk for OIH. Given the acuity of the patient’s distress and an extremely short prognosis of days, the family agreed to have the patient transferred to the hospital to quickly identify the diagnosis and safely rotate the Dilaudid to an alternative opioid as OIH was suspected.

Treatment

In the emergency room (ER) at Kaiser Permanente Los Angeles Medical Center, the patient had a computerized tomography (CT) scan of the brain, which was negative for brain metastasis. His blood work did not show significant metabolic derangement, nor did his vitals show evidence of hypoxia or hypotension. A PICC line nurse assessed his PICC line to be flushing and functioning well to be delivering his IV medications. Rapidly escalating opioid doses without a desired effect of pain relief should alert clinicians to consider other diagnoses including OIH. If OIH is suspected, it is suggested to stop the offending drug. Alternatively, other treatment options are to rotate opioids or wean the current opioid and add medications that antagonize the NMDA receptor such as methadone or ketamine, which create an opioid sparing effect. The inpatient palliative care team was consulted and upon review of the chart and his clinical picture, the team confirmed the suspected diagnosis of OIH.

The treatment plan was to rotate IV Dilaudid to IV methadone. This was discussed extensively with his wife, his durable power of attorney for healthcare, as the patient did not have capacity by this time. His dose was calculated based on his total oral morphine equivalent (OME), which was 46,500 mg in a 24-hour period. The conversion to methadone after intense dose escalation can pose significant problems, as there is no exact reference ratio.

Existing conversion regimens do not address the specific setting of dose escalation and OIH, so a conservative morphine-methadone ratio is recommended. Alternatively, calculating the initial dose of methadone can be based on the opioid usage prior to the pain crisis where rapid escalation was required [3]. Methadone is characterized by extra opioid effects, non-linear pharmacokinetics, and high potential for drug interactions, which makes it difficult to predict appropriate conversion ratios. Based on the patient’s extremely high OME requirements, a conservative 20:1 ratio was taken calculating to methadone 15 mg q3h prn pain. One dose was given in the ER as the IV Dilaudid dose was decreased by 60% from 15 mg/hr to 6 mg/hr. He required three more doses in 12 hours before stabilizing, which equated to 90 mg of IV methadone in a 24 hr period, and he was discharged home two days later on Friday 11/3/17 on IV methadone of 3.5 mg/hr continuous and IV morphine of 30 mg q30min prn breakthrough pain by PCA.

Outcome and follow-up

In less than six hours of being home the patient started developing signs of OIH again with each demand morphine dose and worsening agitation. Since the patient was in his final hours to days of life and had previously stated preferences to die at home, his family declined taking the patient back to the hospital. It was decided to try using IV methadone as a prn breakthrough medication instead of morphine since it logistically was too difficult to get alternative medications such as IV fentanyl or ketamine from the pharmacy on a weekend and delivered to the patient’s home in a timely manner. With minimal data available for reference, prn methadone was conservatively dosed at 5% of his total basal rate requirements, and given the time to peak effect of parenteral methadone is one to two hours, methadone 4 mg IV q1h prn breakthrough pain was prescribed via his CADD pump PCA. This adjustment was made by his visiting home hospice nurse the same day of hospital discharge and explained to his wife who was in agreement with the plan.

On a follow-up phone call later that night, his wife reported that he only required three doses of prn methadone within six hours of the adjustment. She admitted that the first time they pushed the PCA button it was out of fear of pain and agitation rather than actual symptoms. She reported that night was the first night he had slept comfortably in the last several weeks. The next day on 11/4/17, 18 hours after the prn opioid was rotated to methadone, he died peacefully surrounded by loved ones. His family appreciated the hard work and efforts of the entire hospice and palliative medicine teams to honor his wishes of dying at home in peace. The timeline of events is listed in Table 1.

**Table 1:** Timeline of Events

Dates	Patient Milestones
April 2017	Diagnosis of metastatic pancreatic cancer
May 2017	Started on Kaiser home palliative care
September 2017	Transitioned to Kaiser home hospice care
11/2/17-11/3/17	Hospitalization for suspected OIH and safe opioid rotation
11/4/17	Date of death

## Discussion

The management of severe cancer-related pain in a patient with cancer can be complex. OIH is difficult to recognize and, in many cases, may be under-reported as they are often thought to be due to worsening disease, opioid dependence or tolerance [4]. OIH is thought to be mediated by two mechanisms: 1) desensitization of the antinociceptive opioid pathway due to mu-opioid receptor tolerance, and 2) sensitization of the pronociceptive NMDA pathway as a compensatory response to opioid receptor stimulation [5]. This results in increased sensitivity to pain proportional to the dose and strength of the opiate used. NMDA pathway stimulation also causes other forms of neurotoxicity, including cognitive impairment, dysphoria, delirium, hallucinations, myoclonus, and seizures [5]. Other proposed mechanisms include toxic effects of opioid metabolites (M3G, H3G), intracellular protein kinase C, spinal dysmorphin activity, central sensitization of NMDA receptors, and enhanced descending facilitation from the ventromedial medulla.

OIH has been recognized clinically but is under-reported particularly with the misdiagnosis of OIH in comparison to opioid tolerance, withdrawal, and addiction [2]. Animal models have been studied to hypothesize the molecular signaling involved in the phenomenon of OIH, which has been postulated to be the up-regulation of the NMDA receptor antagonist in the dorsal horn neuron; hence the success of drugs such as ketamine in the alleviation of OIH [2].

Methadone is a synthetic high-efficacy opioid agent that is one of the most potent agonists of the mu-opioid receptor, thought to be the main mediator of analgesia. Methadone has the added benefit of blocking the NMDA receptor, which plays a role in neuronal excitotoxicity when activated but can produce dissociative and euphoric effects when blocked. Because of its unique pharmacokinetics, the use of methadone is not only an effective alternative to pure-opioid receptor agonists, but also may be more effective for intractable cancer pain and offer more advantages over morphine or Dilaudid in end-of-life care.

## Conclusions

The unique use of IV methadone by way of continuous infusion and PCA in this case resulted in effective pain control without dose escalation and treated OIH. Methadone should be considered earlier on as a treatment option over other opioids in palliative pain management due to its unique dual mu-opioid receptor agonism and NMDA receptor blockade, no known active metabolites, low cost, high bioavailability, and versatile formulations. Although there are risks associated with methadone such as QT prolongation, variable and long half-life, and drug-drug interactions, these may be acceptable side effects in end-of-life care when comfort is the main priority and other opioids are intolerable. The upregulation of NMDA in OIH and the role of the mu-opioid receptors involved in allodynia and neuropathic pain are still being studied. Further clinical trials will be needed to investigate OIH and the use of methadone in other conditions, especially its off-label use as a prn medication under certain circumstances.
